# Association of Job Expectations Among High School Students With Early Death During Adulthood

**DOI:** 10.1001/jamanetworkopen.2020.27958

**Published:** 2020-12-01

**Authors:** Chandra Muller, Alicia Duncombe, Jamie M. Carroll, Anna S. Mueller, John Robert Warren, Eric Grodsky

**Affiliations:** 1 Population Research Center, Department of Sociology, The University of Texas at Austin; 2 Department of Sociology, Indiana University, Bloomington; 3 Minnesota Population Center, Department of Sociology, University of Minnesota, Minneapolis; 4 Department of Sociology, University of Wisconsin, Madison

## Abstract

**Importance:**

Deaths from self-injury are increasing. Understanding the sources of risk is important for prevention and treatment.

**Objective:**

To estimate the risks of suicide and drug poisoning deaths among adult men whose adolescent occupational expectations were not met in adulthood.

**Design, Setting, and Participants:**

This cohort study included a sample of men interviewed as part of the High School and Beyond study, a nationally representative study of US high school sophomores and seniors in 1980, who were interviewed every 2 years through 1986; those who were sophomores in 1980 were reinterviewed in 1992. Men who survived to 1992 and reported occupational expectations were included in the present study. Death records prior to 2018 were linked to mortality databases and released in 2019. Data analysis was conducted from May to October 2020.

**Exposure:**

Occupational expectations.

**Main Outcomes and Measures:**

Survival or death by suicide, drug poisoning, chronic liver disease, heart disease, cancer, or some other cause, categorized from *International Classification of Diseases, Ninth Revision *and *Tenth Revision* codes. Competing risk Fine-Gray survival models regressed cause of death on adolescent occupational expectations and covariates.

**Results:**

The 11 680 men in the High School and Beyond cohort study had a median (interquartile range) age of 29 (28-30) years in 1992, when the analysis of their future mortality began. Most men survived until 2015 (11 060 [weighted percentage, 95.0%]). Reported causes of death were suicide (60 [weighted percentage, 0.5%]), drug poisoning (40 [weighted percentage, 0.4%]), chronic liver disease (20 [weighted percentage, 0.2%]), heart disease (130 [weighted percentage, 1.0%]), cancer (100 [weighted percentage, 1.0%]), and other (280 [weighted percentage, 2.0%]). Subhazard ratios for death by suicide and drug poisoning were 2.91 (95% CI, 1.07-7.88; *P* = .04) and 2.62 (95% CI, 1.15-5.94; *P* = .02) times higher, respectively, among those who in 1980 expected to hold a subbaccalaureate occupation that later declined in labor market share compared with those with professional occupational expectations. The actual job held by men did not attenuate the hazards of deaths from suicide and drug poisoning.

**Conclusions and Relevance:**

In this cohort study, men whose occupational expectations were not met because of labor market declines were at a higher risk of death from suicide or drug poisoning than men with different occupational expectations. Interventions to mitigate labor market changes should account for individuals’ expectational ideals.

## Key Points

**Question:**

Are unrealized occupational expectations associated with higher risk of suicide?

**Findings:**

In this cohort study of 11 680 male participants who were high school students in 1980, adult deaths from suicide and drug poisoning were more than 2.5 times higher among those who planned for a working-class job if their expected occupation declined in labor market share between 1980 and 1990 compared with those who planned for professional occupations.

**Meaning:**

These findings suggest that policies mitigating the risks from labor market changes must account for the gap between expectational ideals and current labor market opportunities.

## Teaser

This cohort study estimates the risks of suicide and drug poisoning deaths among adult men whose adolescent occupational expectations were not met in adulthood.

## Introduction

Early mortality from self-injury, especially suicide and drug poisoning, has risen dramatically in recent decades, particularly among men without a bachelor’s degree.^1,2,3^ The twin epidemics have cooccurred with 2 trends that suggest despair as a possible common root cause. First, men’s well-paying jobs that do not require a college degree declined during the same period. Fewer jobs, lower wages, and worse benefits contributed to the overall degradation of working-class men’s ability to earn a family wage.^1,4^ Second, early mortality from other causes researchers classify as preventable, such as heart disease and cancer, has steadily declined, in part due to advances in medicine and public health.^5^ In stark contrast, between 1980 and 2013, deaths by suicide and drug poisoning increased by 9 and 31 per 100 000 individuals, respectively, among non-Hispanic White men aged 45 to 55 years.^6^ Combined, the trends suggest that closed pathways to sustaining working-class jobs may contribute to men’s increasing rates of suicide and drug poisoning mortality.^7,8^ This study investigates this possibility with newly available and uniquely suitable data.

Sociologists have long offered such explanations for the so-called evolutionary riddle^9^ of why people would defy a basic survival instinct to bring about their own death through self-injury. Emile Durkheim’s classic work on suicide^10^ argued that societal rates of death from suicide and other forms of self-injury are related to how communities provide individual members with a sense of purpose that makes life worth living, even in times of stress. Work plays a major role in how individuals experience their communities,^11^ derive a sense of purpose, and thus develop a sense of psychological well-being.^12,13,14^ When men’s desired and expected lines of work decline, they may confront a crisis of confidence and identity that heightens their risk of self-injury and death. As sociologist Robert Merton argued, individuals who embrace cultural ideals for success, such as expectations to hold a good job as an adult, to serve others in the community, or to support their family, might resort to suicide as an escape without an institutionally acceptable pathway to success.^15,16^ We investigated whether the relative decline of working-class, subbaccalaureate occupations was associated with an increased risk of death from suicide and drug poisoning among men who expected to hold such jobs when they were in high school.

Many men now in the high-risk age group for death from self-injury were in high school in the early 1980s and reasonably expected to go into lines of work that could support a middle-class lifestyle without a college degree. Many of those lines of work subsequently declined with labor market changes. Adolescents form and internalize expectations about their future social roles that may serve as an ideal or benchmark for self-perceived success during adulthood.^17^ The labor market changes generated broad-based challenges to finding a stable, well-paying job for men who did not obtain a college degree, blocking pathways to fulfilling their ideals. We hypothesized that unmet occupational plans could contribute to despair and consequently be associated with increases in the risk of death from suicide or drug poisoning.

Although despair has been widely speculated as a source of self-injury deaths,^18^ until now we have lacked data to empirically evaluate an association between (possibly unrealized) early life expectations and so-called deaths of despair and instead relied on indicators of college degree attainment as a risk factor.^7^ Identifying unmet expectations as a risk factor for suicide and drug poisoning deaths many years later is challenging because expectations are products of school experiences and childhood resources (such as growing up with more educated parents) that could also affect mortality risk through educational attainment. Moreover, educational degrees sort individuals into work, marriage, access to health care, and other social institutions that structure pathways through adulthood and to mortality outcomes.^19^ Our study design used data collected from students during their formative high school years and early adulthood matched to death records to identify the early life experiences that are associated with early adult mortality. With this clarity, we can begin to pinpoint whether unmet occupational expectations are associated with the risks of death from self-injury or other causes. An etiologic understanding of these early deaths is essential for prevention and treatment.

## Methods

### Sample

We used data from the High School and Beyond cohort, a nationally representative sample of sophomores (approximately 14 830 participants, with approximately 7350 [49.6%] male participants) and seniors (approximately 12 000 participants, with approximately 5680 [47.3%] male participants) attending more than 1000 public and private (including religious) high schools across the United States in 1980. All sample sizes have been rounded to the nearest 10 to comply with restricted-use data reporting requirements. Sample members were mostly born between 1961 and 1965 and were reinterviewed every 2 years until 1986. Participants who were sophomores in 1980 were interviewed once more in 1992 at age approximately 28 years. Follow-up response rates were very high (23 970 [weighted percentage, 94.0%] in 1982, 23 350 [weighted percentage, 91.0%] in 1984, 22 720 [weighted percentage, 88.0%] in 1986, and 11 850 [weighted percentage, 85.0%] in 1992), with 19 900 (weighted percentage, 79.0%) participating in all waves. Sample members were reinterviewed at midlife, in 2014 to 2015, and mortality status was ascertained in 2017 for nonrespondents by matching records to National Death Index and the Social Security Death Index, augmented by internet searches, genealogical websites, credit bureau databases, and online obituaries.^20,21^ Our analysis included men who survived to 1992, when most were aged between 27 and 31 years. Results are robust to defining survival to age 27. We further excluded respondents for whom we lacked a report of occupational expectations and those who did not expect to work at age 30 years; the final analytic sample included approximately 11 680 participants. The study using deidentified data was approved by the University of Texas institutional review board. Respondents provided consent during data collection by the US Department of Education, National Center for Education Statistics. This study followed the Strengthening the Reporting of Observational Studies in Epidemiology (STROBE) reporting guideline.

### Measures

Our dependent variable was survival until 2015 vs death, by cause, with the aim of modeling a source of self-injury deaths. We followed Masters et al^6^ and used the *International Statistical Classification of Diseases and Related Health Problems, Ninth Revision* (*ICD*-*9*) and *Tenth Revision* (*ICD*-*10*) to identify the following 6 reported cause-of-death categories: suicide, drug poisoning, chronic liver disease, heart disease, cancer, or another cause. The first 3 categories have been called deaths of self-injury or despair.^2,7,11^ We focused on suicide and drug poisoning, which had large enough incidences in our sample for statistical analysis. We also singled out 2 common causes of early mortality that researchers classify as preventable, heart disease and cancer, that have declined during the same period that self-injury deaths have increased.^6^ Using the year of death, our modeling strategy takes account of years of survival after 1992.

#### Occupational Expectations

In their senior year of high school, boys were asked, “What kind of work will you be doing when are you 30 years old?” In 1982, when individuals who entered the study as sophomores were in their senior years, they were asked, “Write in here the name of the job or occupation that you expect or plan to have when you are 30 years old. … Which of the categories below comes closest to describing that job?” They chose from 15 occupation groups that we collapsed into the following 3 categories: (1) occupations that mostly did not require a college degree (ie, subbaccalaureate) that lost labor market share (LMS) between 1980 and 1990, (2) subbaccalaureate occupations that gained LMS or were stable, and (3) professional occupations (which all increased in LMS) requiring a college degree (reference group). The 15 occupational groups and their LMS attributes are shown in eTable 1 in the Supplement. To calculate the change in LMS, we used the 1980 and 1990 US Census data (5% state, individual-level samples) from a publicly available data portal managed by Integrated Public Use Microdata Series–USA.^22^ After restricting the samples to those who were employed in each census year, there were approximately 4.9 million and 5.8 million workers from the 1980 and 1990 census, respectively. Two independent researchers (A.D. and a second researcher) matched each census occupation category to 1 of the 14 High School and Beyond occupation categories (excluding proprietors, for which there was no equivalence in the census occupations), and differences were discussed, reconciled, and verified by a third researcher (J.M.C.). The percentage growth (or decline) of the LMS is the difference between the 1980 and 1990 percentages of LMSs for each of the 14 categories divided by the LMS of 1980.

#### School Experiences and Background

Academic experiences were measured by the highest level of mathematics course the student completed by the end of high school (ie, took Algebra I and/or more advanced course [reference group] vs did not). In the early 1980s, not completing Algebra I indicated that the student was unprepared for college. We also controlled for students’ achievement test scores (composite of math and verbal test scores; range, 27.41-71.36) measured in the senior year. Nonacademic experiences, all self-reported, included (1) not attending church weekly (reference group, attending church weekly or more); (2) having overweight (reference group, not having overweight); (3) having any depressive thoughts in the prior month (reference group, no depressive thoughts in prior month); and (4) locus of control, a scale that indicates the extent to which students believe they can influence their own life outcomes (range, −3.03 to 1.27, with higher score indicating greater control).

We categorized parents’ highest level of education into the following 3 categories: at least 1 parent completed a high school diploma or less (reference group), at least 1 parent had some postsecondary education, or at least 1 parent had at least a bachelor degree. Race/ethnicity was categorized as non-Hispanic White (reference), non-Hispanic Black, Hispanic (regardless of race), or other. Students reported their sex and race/ethnicity, and when missing, it was determined from other sources (eg, high school report). Schools’ urbanicity categorized as was urban, suburban, or rural (reference group).

#### Educational Attainment

We also controlled for highest educational degree attained to estimate the hazard of occupational expectations as independent from educational attainment. Postsecondary transcripts, collected in 1986 (among seniors) and 1992 (among sophomores), and supplemented with self-reports provided highest level of education, categorized as less than high school; high school diploma (reference group); some postsecondary education or postsecondary education that did not culminate in a 4-year degree; or bachelor’s degree or more.

#### Early Adulthood Occupation

In 1986 (among seniors) and 1992 (among sophomores), respondents reported their current or most recent occupation. Categories were harmonized to categories of occupational expectations.

### Statistical Analysis

All statistical analyses were performed in Stata version 15 (StataCorp). We estimated 2 separate sets of competing-risk survival models of deaths between 1993 and 2015, adjusted for observable, time-constant, and confounding variables.^23^ We present exponentiated regression coefficients from Fine-Gray subdistribution hazard regression models, which show the rates of death for each cause of death relative to being alive or dying from another cause.^24^ We show *P* values for all coefficients. To test our main hypothesis about the association between high school occupational expectations and hazard of deaths from self-injury or despair in adulthood, our first competing risk models regress the subdistribution hazard (or subhazard) of each cause of death on occupational expectations while in high school. To isolate occupational expectations as a risk in self-injury deaths, high school experiences, background, and educational attainment were also included as controls in the models. Our second models included the job men held in early adulthood to consider the possibility that it is associated with deaths of despair.

Because of the complex sampling design of the High School and Beyond study, we weight all of our analyses so that our estimates are generalizable to male sophomores and seniors enrolled in US high schools in 1980 who survived to 1992. We clustered the standard errors at the school level. We used multiple imputation (30 imputations) for all variables except those used as sample filters (mortality status and occupational expectations). The multiple imputation allowed us to estimate unbiased parameters of interest while retaining sample size. The proportion of missing values for each variable is shown in Table 1. Statistical significance was set at *P* < .05, and all tests were 2-tailed.

**Table 1.** Descriptive Statistics by Mortality and Cause of Death, High School and Beyond Sample^a^

Characteristic	Alive	Suicide	Drug poisoning	Disease		Cancer	Other	PM
				Chronic liver	Heart			
No. (weighted percentage)	11 060 (95.0)	60 (0.5)	40 (0.4)	20 (0.2)	130 (1.0)	100 (1.0)	280 (2.0)	NA
Age at death, median (IQR), y	NA	42 (36-48)	46 (43-50)	47 (45-50)	46 (41-51)	46 (42-50)	42 (36-48)	NA
Occupational expectations								
Subbaccalaureate								0
Declining	0.33	0.53	0.52	0.36	0.46	0.31	0.39	
Increasing	0.17	0.16	0.13	0.27	0.14	0.17	0.15	
Professional	0.50	0.30	0.34	0.37	0.40	0.52	0.46	
No advanced math	0.21	0.35	0.42	0.06	0.35	0.43	0.35	.03
Test scores, mean (SD)	51 (9)	52 (10)	49 (9)	50 (11)	47 (8)	49 (9)	47 (8)	.10
Church attendance	0.40	0.44	0.19	0.44	0.35	0.31	0.38	.14
Overweight	0.12	0.08	0.12	0.14	0.26	0.21	0.17	.04
Depressive thoughts	0.55	0.73	0.78	0.53	0.55	0.50	0.61	.09
Locus of control, mean (SD)	−.04 (.68)	−.07 (.75)	−.35 (.70)	-.03 (.51)	−.09 (.66)	−.07 (.74)	−.16 (.68)	.07
Parents’ highest education								
≤High school	0.40	0.45	0.19	0.17	0.45	0.46	0.51	.04
Some college or vocational school	0.30	0.30	0.40	0.16	0.28	0.40	0.28	
≥Bachelor degree	0.30	0.25	0.41	0.67	0.27	0.14	0.21	
Race								
Non-Hispanic								0
White	0.75	0.80	0.69	0.58	0.69	0.76	0.56	
Black	0.11	0.09	0.10	0.06	0.18	0.10	0.16	
Hispanic	0.12	0.09	0.20	0.32	0.11	0.09	0.22	
Other	0.03	0.02	0.01	0.03	0.02	0.05	0.06	
Urbanicity								
Urban	0.20	0.22	0.12	0.20	0.22	0.21	0.29	0
Suburban	0.49	0.47	0.73	0.30	0.37	0.42	0.42	
Rural	0.31	0.31	0.15	0.50	0.41	0.37	0.29	
Educational attainment								
<High school	0.03	0.04	0.11	0.06	0.07	0.04	0.11	.02
High school diploma	0.31	0.30	0.23	0.37	0.36	0.39	0.36	
Some college or vocational school	0.43	0.57	0.51	0.42	0.49	0.48	0.49	
≥Bachelor degree	0.22	0.08	0.15	0.15	0.08	0.09	0.05	
Early adult occupation								
Subbaccalaureate								
Declining	0.50	0.66	0.43	0.66	0.55	0.52	0.58	.10
Increasing	0.27	0.11	0.17	0.34	0.16	0.31	0.19	
Professional	0.22	0.23	0.39	0^b^	0.29	0.17	0.23	
Member of senior cohort	0.40	0.35	0.47	0.52	0.47	0.42	0.45	0

Abbreviations: IQR, interquartile range; NA, not applicable; PM, proportion missing from whole sample.

^a^ US Department of Education, National Center for Education Statistics, High School and Beyond Panel Sample Men who survived to 1992, excluding those who had no occupational expectations (1010 [13.2%]) and those who expected not to work at age 30 (140 [1.0%]), matched to mortality records.

^b^ Among those who died of chronic liver disease, none worked in a professional occupation in early adulthood.

## Results

The 11 680 men in this study had a median (interquartile range) age of 29 (28-30) years in 1992, when the analysis of their future mortality began. Descriptive statistics for mortality and all other variables are shown in Table 1. Overall, most participants alive in 1992 survived during the study period until 2015 (11 060 [weighted percentage, 95.0%]), with less than 6% of the sample having died by any cause. Distributions for the independent variables by each cause of death are compared with the distributions among surviving sample members using bivariate logistic or ordinary least squares regression. *P* values from those analyses are reported in eTable 2 in the Supplement. A relatively small share of the sample had died by either suicide (approximately 60 participants [weighted percentage, 0.5%]) or drug poisoning (approximately 40 participants [weighted percentage, 0.4%]), with even fewer deaths from chronic liver disease (approximately 20 participants [weighted percentage, 0.2%]). More sample members had died from heart disease (approximately 130 participants [weighted percentage, 1.0%]), cancer (approximately 100 participants [weighted percentage, 1.0%]), or other causes (approximately 280 participants [weighted percentage, 2.0%]).

The Figure shows the cumulative percentage of deaths from each cause of death each year from 1993 to 2015. Death by suicide and drug poisoning occurred during the early to middle adulthoods of these men at a fairly steady rate. The mortality risk due to heart disease and cancer increased gradually over adulthood, as did deaths due to all other causes.

**Figure.**
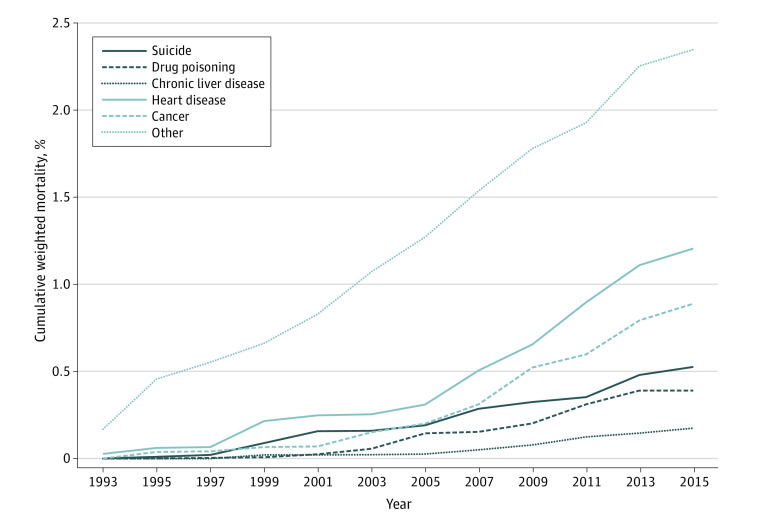
Cumulative Weighted Percentages of Mortality by Cause of Death and Year

Table 2 shows subhazard ratios from a competing-risk regression model of cause of death (relative to survival) regressed on early life occupational expectations, school and adolescent experiences, and background. Of primary interest are the ratios of deaths for men who held different occupational expectations when they were in high school. Deaths by suicide and drug poisoning were 2.91 (95% CI, 1.07-7.88; *P* = .04) and 2.62 (95% CI, 1.15-5.94; *P* = .02) times higher, respectively, among high school boys who expected to hold a subbaccalaureate occupation that later declined in LMS compared with those who held professional occupational expectations. Occupational expectations were not associated with mortality by other causes.

**Table 2.** Association Between Adolescent Occupational Expectations and Dying by Suicide or Drug Poisoning in Adulthood Among 11 680 Participants in the High School and Beyond Study^a^

Variable	Suicide		Drug poisoning		Chronic liver disease		Heart disease		Cancer		Other disease	
	Subhazard ratio (95% CI)	*P* value	Subhazard ratio (95% CI)	*P* value	Subhazard ratio (95% CI)	*P* value	Subhazard ratio (95% CI)	*P* value	Subhazard ratio (95% CI)	*P* value	Subhazard ratio (95% CI)	*P* value
Subbaccalaureate occupational expectation												
Decreasing	2.91 (1.07-7.88)	.04	2.62 (1.15-5.94)	.02	1.49 (0.39-5.74)	.56	1.19 (0.62-2.27)	.60	0.52 (0.26-1.04)	.07	0.82 (0.55-1.24)	.35
Increasing	1.51 (0.51-4.43)	.45	1.22 (0.35-4.23)	.76	2.40 (0.54-10.71)	.45	0.89 (0.43-1.82)	.75	0.75 (0.37-1.55)	.44	0.80 (0.46-1.40)	.44
No advanced math	2.29 (0.98-5.36)	.06	3.16 (1.05-9.50)	.04	0.32 (0.05-1.94)	.21	1.26 (0.66-2.43)	.48	2.97 (1.47-5.99)	.002	1.34 (0.85-2.13)	.21
Test scores	1.06 (0.998-1.12)	.06	1.03 (0.95-1.12)	.48	0.99 (0.89-1.09)	.80	0.97 (0.93-1.01)	.16	1.01 (0.96-1.06)	.68	0.99 (0.96-1.03)	.71
Church attendance	1.29 (0.65-2.53)	.46	0.50 (0.16-1.54)	.23	1.07 (0.31-3.64)	.91	0.93 (0.51-1.70)	.83	0.86 (0.48-1.54)	.61	1.00 (0.64-1.54)	.99
Overweight	0.61 (0.17-2.24)	.46	0.90 (0.26-3.13)	.87	1.22 (0.20-7.36)	.83	2.58 (1.42-4.69)	.002	1.77 (0.89-3.54)	.11	1.40 (0.87-2.27)	.17
Depressive thoughts	2.11 (0.95-4.67)	.07	2.15 (0.70-6.61)	.18	0.95 (0.30-3.01)	.93	0.81 (0.45-1.47)	.49	0.76 (0.44-1.31)	.32	1.11 (0.71-1.73)	.64
Locus of control	1.10 (0.54-2.24)	.80	0.60 (0.32-1.12)	.11	1.13 (0.64-1.97)	.68	1.19 (0.76-1.85)	.46	1.14 (0.71-1.83)	.60	1.06 (0.78-1.44)	.71
Parents’ highest education												
Some college	0.90 (0.40-2.04)	.80	3.53 (1.30-10.00)	.02	1.71 (0.35-8.47)	.51	0.99 (0.57-1.71)	.96	1.17 (0.65-2.11)	.61	0.80 (0.52-1.25)	.34
≥Bachelor degree	0.89 (0.34-2.28)	.80	5.33 (1.77-16.08)	.003	10.48 (2.42-45.43)	.002	1.31 (0.68-2.56)	.42	0.51 (0.22-1.20)	.12	0.80 (0.50-1.29)	.36
Race												
Black, non-Hispanic	0.84 (0.33-2.13)	.71	1.22 (0.26-5.62)	.80	0.96 (0.20-4.66)	.96	1.46 (0.78-2.73)	.24	0.73 (0.34-1.59)	.43	1.43 (0.89-2.30)	.14
Hispanic	0.66 (0.29-1.54)	.34	1.95 (0.76-5.01)	.17	4.57 (1.59-13.07)	.005	0.76 (0.36-1.61)	.48	0.61 (0.32-1.16)	.13	1.88 (1.17-3.02)	.009
Other	0.74 (0.10-5.47)	.77	0.30 (0.05-1.70)	.18	1.72 (0.27-10.98)	.57	0.76 (0.32-1.79)	.53	1.50 (0.30-7.45)	.62	2.37 (0.85-6.62)	.10
Urbanicity												
Urban	1.45 (0.58-3.65)	.43	1.19 (0.28-5.18)	.81	0.50 (0.09-2.69)	.42	0.79 (0.40-1.57)	.51	0.87 (0.38-1.98)	.74	1.31 (0.76-2.23)	.33
Suburban	1.13 (0.53-2.44)	.75	3.33 (1.16-9.60)	.03	0.31 (0.07-1.31)	.11	0.63 (0.33-1.20)	.16	0.77 (0.41-1.45)	.42	1.02 (0.65-1.60)	.92
Educational attainment												
<High school	1.20 (0.23-6.30)	.83	3.83 (0.80-18.27)	.09	2.50 (0.39-15.84)	.33	1.66 (0.40-6.83)	.48	0.85 (0.32-2.27)	.75	2.25 (0.97-5.25)	.06
Some college	1.69 (0.77-3.70)	.19	2.01 (0.75-5.39)	.16	0.54 (0.11-2.68)	.45	1.14 (0.61-2.13)	.69	1.02 (0.55-1.90)	.95	1.06 (0.69-1.62)	.79
≥Bachelor degree	0.50 (0.10-2.44)	.39	1.71 (0.32-9.28)	.53	0.38 (0.03-4.48)	.44	0.51 (0.17-1.54)	.23	0.44 (0.14-1.35)	.15	0.29 (0.12-0.69)	.005
Member of senior cohort	0.79 (0.39-1.61)	.52	1.54 (0.66-3.61)	.32	1.80 (0.52-6.20)	.35	1.39 (0.86-2.25)	.18	1.15 (0.63-2.11)	.65	1.41 (1.06-1.89)	.02

^a^ US Department of Education, National Center for Education Statistics, High School and Beyond Panel Sample including men who survived to 1992, excluding those who had no occupational expectations and those who expected not to work at age 30, matched to mortality records.

To test whether the job held in early adulthood was associated with risk of early death, we included the self-reported occupation type for the job the respondent held in early adulthood in a model with the other variables from Table 2. The subhazard ratios of mortality by suicide or drug poisoning were not associated with the occupational sector of the men’s actual jobs (Table 3). Importantly, early adulthood occupational sector did not attenuate the significant coefficients indicating heightened suicide and drug poisoning hazards among men who expected a working-class occupation that subsequently declined in LMS. Rather, the men who expected such an occupation had approximately 2.73 (95% CI, 1.02-7.30; *P* = .045) and 2.76 (95% CI, 1.18-6.46; *P* = .02) times higher hazards of death from suicide or drug poisoning, respectively, even when their actual occupation was held constant.

**Table 3.** Association of Occupational Attainment With Dying of Suicide or Drug Poisoning in Adulthood Among 11 680 Participants in the High School and Beyond Study^a^

Variable	Suicide		Drug poisoning		Chronic liver disease		Heart disease		Cancer		Other disease	
	Subhazard ratio (95% CI)	*P* value	Subhazard ratio (95% CI)	*P* value	Subhazard ratio (95% CI)	*P* value	Subhazard ratio (95% CI)	*P* value	Subhazard ratio (95% CI)	*P* value	Subhazard ratio (95% CI)	*P* value
Subbaccalaureate occupational expectation												
Decreasing	2.73 (1.02-7.30)	.045	2.76 (1.18-6.46)	.02	1.45 (0.34-6.15)	.61	1.21 (0.65-2.23)	.55	0.54 (0.26-1.11)	.09	0.83 (0.55-1.25)	.36
Increasing	1.45 (0.49-4.26)	.50	1.13 (0.32-4.00)	.85	2.65 (0.60-11.62)	.19	0.86 (0.42-1.75)	.68	0.78 (0.38-1.59)	.49	0.80 (0.46-1.40)	.43
No advanced math	2.20 (0.95-5.13)	.07	3.21 (1.04-9.95)	.04	0.32 (0.05-1.95)	.21	1.27 (0.66-2.43)	.48	3.07 (1.52-6.20)	.002	1.34 (0.84-2.14)	.22
Test scores	1.06 (0.998-1.12)	.06	1.03 (0.95-1.12)	.49	0.99 (0.89-1.10)	.85	0.97 (0.93-1.01)	.15	1.01 (0.96-1.06)	.69	0.99 (0.96-1.03)	.71
Church attendance	1.28 (0.65-2.51)	.48	0.50 (0.16-1.57)	.23	1.08 (0.32-3.66)	.91	0.93 (0.51-1.70)	.81	0.87 (0.49-1.55)	.63	1.00 (0.64-1.54)	.99
Overweight	0.60 (0.16-2.19)	.44	0.88 (0.25-3.11)	.84	1.20 (0.19-7.44)	.85	2.58 (1.41-4.72)	.002	1.79 (0.89-3.59)	.10	1.40 (0.87-2.26)	.17
Depressive thoughts	2.13 (0.97-4.71)	.06	2.14 (0.70-6.56)	.18	0.96 (0.30-3.09)	.95	0.81 (0.44-1.47)	.48	0.76 (0.44-1.30)	.31	1.11 (0.71-1.73)	.64
Locus of control	1.10 (0.54-2.22)	.79	0.60 (0.32-1.11)	.10	1.16 (0.66-2.02)	.61	1.18 (0.75-1.84)	.48	1.13 (0.70-1.83)	.61	1.06 (0.78-1.44)	.72
Parents’ highest education												
Some college	0.91 (0.40-2.05)	.82	3.49 (1.23-9.91)	.02	1.70 (0.35-8.35)	.52	0.99 (0.57-1.72)	.97	1.17 (0.65-2.11)	.61	0.80 (0.52-1.25)	.34
≥Bachelor’s or more	0.90 (0.35-2.32)	.82	5.12 (1.68-15.59)	.004	10.50 (2.54-43.20)	.001	1.31 (0.67-2.56)	.43	0.51 (0.22-1.20)	.12	0.80 (0.49-1.29)	.36
Race												
Black, non-Hispanic	0.80 (0.31-2.05)	.64	1.14 (0.24-5.39)	.87	1.08 (0.22-5.43)	.92	1.41 (0.76-2.63)	.27	0.77 (0.35-1.68)	.51	1.42 (0.89-2.29)	.14
Hispanic	0.65 (0.28-1.51)	.32	1.92 (0.75-4.96)	.18	4.77 (1.71-13.33)	.003	0.75 (0.36-1.58)	.46	0.62 (0.32-1.17)	.14	1.88 (1.17-3.01)	.009
Other	0.73 (0.10-5.52)	.76	0.31 (0.05-1.72)	.18	1.71 (0.26-11.36)	.58	0.75 (0.32-1.77)	.51	1.50 (0.30-7.44)	.62	2.37 (0.85-6.63)	.10
Urbanicity												
Urban	1.46 (0.58-3.67)	.42	1.12 (0.25-5.00)	.89	0.51 (0.10-2.57)	.42	0.78 (0.40-1.55)	.48	0.87 (0.38-1.99)	.74	1.30 (0.77-2.21)	.33
Suburban	1.15 (0.54-2.46)	.72	3.16 (1.08-9.30)	.04	0.32 (0.08-1.33)	.12	0.62 (0.32-1.17)	.14	0.77 (0.41-1.45)	.42	1.02 (0.65-1.59)	.93
Educational attainment												
<High school	1.17 (0.22-6.13)	.85	3.48 (0.69-17.59)	.13	2.79 (0.42-18.68)	.29	1.62 (0.40-6.67)	.50	0.88 (0.33-2.36)	.80	2.24 (0.97-5.20)	.06
Some college	1.74 (0.80-2.81)	.16	1.92 (0.66-5.60)	.24	0.64 (0.14-3.03)	.58	1.10 (0.57-2.13)	.77	1.01 (0.54-1.92)	.97	1.06 (0.69-1.61)	.80
≥Bachelor degree	0.60 (0.12-3.00)	.53	1.66 (0.26-10.38)	.59	0.40 (0.02-7.00)	.53	0.51 (0.15-1.69)	.27	0.39 (0.13-1.15)	.09	0.29 (0.12-0.70)	.006
Early adult subbaccalaureate occupation												
Decreasing	2.08 (0.57-7.66)	.27	1.10 (0.33-3.65)	.88	0.94 (0.20-4.42)	.93	1.09 (0.43-2.74)	.86	0.65 (0.34-1.27)	.21	1.03 (0.64-1.67)	.89
Increasing	2.00 (0.49-8.12)	.33	2.24 (0.68-7.39)	.19	0.00 (0.00-188.46)	.24	1.59 (0.63-4.02)	.33	0.55 (0.27-1.13)	.10	1.10 (0.62-1.95)	.74
Member of senior cohort	0.77 (0.37-1.59)	.47	1.46 (0.64-3.35)	.37	1.95 (0.58-6.55)	.28	1.35 (0.84-2.18)	.22	1.18 (0.65-2.16)	.59	1.41 (1.05-1.89)	.02

^a^ US Department of Education, National Center for Education Statistics, High School and Beyond Panel Sample including men who survived to 1992, excluding those who had no occupational expectations and those who expected not to work at age 30, matched to mortality records.

## Discussion

Although many scholars have speculated that increases in suicide and drug poisoning deaths among men without a college degree are rooted in distress from the decline of working-class jobs, the results we report here are the first, to our knowledge, that empirically show that association. Adolescent boys who expected an occupation at age 30 that subsequently declined in LMS had higher risks of death by suicide and drug poisoning compared with boys who expected to hold a professional job. We underscore 2 aspects of these findings. First, our unique data allowed us to estimate the risk of unmet occupational expectations independently from risk of low educational attainment or actual job in a declining occupation. The semiskilled jobs that placed these men at risk were in the middle of the wage distribution—ie, jobs with wages that could support a family when our sample was growing up—and declined during their adulthoods. The heightened hazards of their unmet expectations were statistically independent of educational attainment and the actual job that men held in early adulthood. Second, occupational expectations were not associated with higher risk of death from heart disease or cancer, 2 other preventable causes of death that do not involve self-injury. These findings are consistent with theories about the distress or despair associated with blocked pathways to achieving self-perceived success. Our results are consistent with our hypothesis that men have a heightened suicide and drug poisoning risk if their occupational expectations are unmet because of a decline in LMS.^25^

It is possible that men who secured a job in the declining subbaccalaureate sector of the labor market were at risk, regardless of the occupational expectations they held in high school. If this were the case, then holding such a job in early adulthood would be associated with suicide or drug poisoning independently from the occupational expectations held in adolescence. Alternatively, it is possible that men who were able to fulfill their occupational expectations, even if the occupational status was in decline, were better off than those who were unable to secure a job in their expected occupational sector. In this case, the actual job held in early adulthood would attenuate the estimated risk of adolescents’ occupational expectations. However, our second model showed that actual job in early adulthood was not associated with increased risk of death from suicide or drug poisoning.

Others have suggested the possibility that other workforce changes may create distress and despair that place workers at risk. For example, several recent articles^26,27^ suggest such risks for health care professionals. These possibilities are worth future empirical study. The social, psychological, and cultural ideals associated with certain occupations are important considerations in labor policy, such as minimum wage policies^28^ or job retraining programs, as strategies for suicide prevention.^29^

### Limitations

This study has limitations. With observational data, our findings should be viewed as associational and descriptive rather than causal. We faced 2 significant challenges in estimating the sources of early adult suicide and drug poisoning. First, even in our sample of nearly 12 000 men, the incidence of death from suicide and drug poisoning was relatively low. Our modeling strategy required parsimonious models while also recognizing that background and school experiences shape both occupational expectations and mortality risk. Furthermore, we sought to estimate the risk from occupational expectations not from attained education and occupation. Nonetheless, the High School and Beyond study is, to our knowledge, the best available nationally representative US data set with an adequate sample size that extends from adolescence to midlife and includes causes of death and prospective information from adolescence and early adulthood. The association between occupational expectations and self-injury deaths is noteworthy.

## Conclusions

This study found an association between unmet occupational expectations and early mortality from suicide and drug poisoning. The findings are consistent with the possibility that occupational expectations developed in adolescence serve as a benchmark for perceptions of adult success and, when unmet, pose a risk of self-injury.

## Notes

Supplement. **eTable 1. **Occupation Categories and Change in Labor Market Share and Wage Percentile Rank Between 1980 and 1990 **eTable 2. ***P* Values for Logistic and Ordinary Least Squares Regression Significance Testing of Differences in Descriptive Statistics
