# Supplementary material for: Association of Job Expectations Among High School Students With Early Death During Adulthood
Source: JAMA Netw Open. 2020 Dec 1;3(12):e2027958. doi: 10.1001/jamanetworkopen.2020.27958 (PMC7709084; doi:10.1001/jamanetworkopen.2020.27958)
Supplement: Supplement. — eTable 1. Occupation Categories and Change in Labor Market Share and Wage Percentile Rank Between 1980 and 1990 eTable 2. P Values for Logistic and Ordinary Least Squares Regression Significance Testing of Differences in Descriptive Statistics [file jamanetwopen-e2027958-s001.pdf]

Supplemental Online Content

Muller C, Duncombe A, Carroll JM, Mueller AS, Warren JR, Grodsky E. Association of job expectations among high school students with early death during adulthood. *JAMA Netw Open*. 2020;3(12):e2027958. doi:10.1001/jamanetworkopen.2020.27958

eTable 1. Occupation Categories and Change in Labor Market Share and Wage Percentile Rank Between 1980 and 1990

eTable 2. *P* Values for Logistic and Ordinary Least Squares Regression Significance Testing of Differences in Descriptive Statistics

This supplemental material has been provided by the authors to give readers additional information about their work.

© 2020 Muller C et al. *JAMA Network Open.*

eTable 1. Occupation Categories and Change in Labor Market Share and Wage Percentile Rank Between 1980 and 1990

Analytic Occupational Categories	*HS&B Questionnaire Categories*	*% of HS&B Sample[a]*	*Most Common Occupations*	*% Male Workers[b]*	*% Change in Labor Market Share[b]*	*Wage Percentile Change[b]*
**Declining**	Clerical	1.24%	Secretary, Shipping Clerk	21.30%	-5.50%	2.42
(Subbacalaureate)	Craftsman	14.08%	Carpenter, Mechanic	93.80%	-7.90%	-3.92
	Farmer, Farm manager	2.77%	Farmer (owner)	90.30%	-9.40%	-0.68
	Laborer	3.20%	Farmer laborer, Construction	82.50%	-8.50%	-1.92
	Military	3.98%	Military	91.10%	-15.40%	N/A
	Operative	5.34%	Drivers, Machine operator	70.40%	-17.20%	-5.90
**Increasing**	Protective	3.06%	Police, Security	88.60%	11.60%	5.83
(Subbacalaureate)	Service	0.69%	Janitor, Waitress/Waiter	35.90%	0.50%	0.23
	Technical	12.36%	Drafter, Technician	59.40%	16.30%	2.83
	Sales	1.70%	Salesperson	48.40%	3.30%	7.79
**Professional**	Manager, admin	7.62%	Manager, Administrator	73.90%	2.30%	3.60
(Baccalaureate)	Professional 1	25.23%	Accountant, Nurse	54.10%	16.30%	5.66
	Professional 2	11.98%	Lawyer, Doctor	75.30%	21.80%	1.45
	School Teacher	1.36%	Primary School Teacher	29.20%	5.30%	2.83
	Proprietor	5.39%	N/A	N/A	N/A	N/A

[a]SOURCE: US Department of Education, National Center for Education Statistics, High School & Beyond (HS&B) Panel Sample Men Matched to Mortality Records
[b]SOURCE: 1980 & 1990 American Community Survey, working population

© 2020 Muller C et al. *JAMA Network Open.*

Table 2. P-values for Logistic and OLS regression significance testing of differences in descriptive statistics (Table 1)

	P-Values from comparison to distribution of those who survived to midlife					
			Disease			
	Suicide	Drug Poisoning	Chronic Liver	Heart	Cancer	Other
Occupational Expectations						
Subbaccalaureate						
Declining	0.02	0.04	0.81	0.02	0.73	0.20
Increasing	0.88	0.63	0.42	0.45	0.88	0.55
Professional	0.03	0.13	0.42	0.10	0.67	0.42
No Advanced Math	0.05	0.01	0.06	0.007	0.000	0.001
Test Scores[a]	0.65	0.37	0.72	0.000	0.18	0.000
Church Attendance	0.70	0.04	0.83	0.37	0.14	0.66
Overweight	0.59	0.97	0.81	0.001	0.04	0.07
Depressive Thoughts	0.24	0.67	0.99	0.75	0.78	0.05
Locus of Control[a]	0.85	0.04	0.93	0.56	0.71	0.07
Parents' Highest Education						
High School	0.53	0.03	0.08	0.43	0.34	0.02
Some College or Vocational School	0.95	0.32	0.18	0.69	0.14	0.62
Bachelor’s degree	0.55	0.23	0.005	0.63	0.009	0.02
Race						
Non-Hispanic						
White	0.37	0.45	0.20	0.28	0.75	0.000
Black	0.69	0.89	0.40	0.03	0.80	0.02
Hispanic	0.39	0.14	0.04	0.76	0.25	0.001
Other	0.81	0.22	0.83	0.56	0.48	0.14
Urbanicity						
Urban	0.77	0.30	0.98	0.62	0.90	0.03
Suburban	0.83	0.02	0.22	0.06	0.33	0.16
Rural	0.99	0.08	0.19	0.15	0.37	0.59
Educational Attainment						
High School	0.73	0.04	0.46	0.20	0.86	0.004
High School Diploma	0.91	0.37	0.66	0.46	0.18	0.28
Some College or Vocational School	0.11	0.45	0.90	0.40	0.49	0.27
Bachelor’s degree	0.09	0.44	0.66	0.009	0.02	0.000
Early Adult Occupation						
Subbaccalaureate						
Declining	0.10	0.52	0.28	0.49	0.75	0.12
Increasing	0.96	0.07	- [b]	0.25	0.30	0.83
Professional	0.05	0.25	0.63	0.10	0.59	0.03
Member of Senior Cohort	0.52	0.54	0.39	0.21	0.79	0.28

SOURCE: U.S. Department of Education, National Center for Education Statistics, High School & Beyond Panel Sample Men who survived to 1992, excluding those who had no occupational expectations and those who expected not to work at age 30, matched to mortality records
Abbreviations: DP, drug poisoning; CL, chronic liver
[a] modeled with OLS regression; [b] Among those who died by chronic liver disease, none worked in a professional occupation in early adulthood

© 2020 Muller C et al. *JAMA Network Open.*
